# Comparative analysis of arthroscopic-assisted Tight-rope technique and clavicular hook plate fixation in the treatment of Neer type IIB distal clavicle fractures

**DOI:** 10.1186/s12891-022-05724-9

**Published:** 2022-08-06

**Authors:** Si Nie, Hong-Bo Li, Li Hua, Zhi-Ming Tang, Min Lan

**Affiliations:** 1 Department of Radiology, Jiangxi Provincial People’s Hospital, (The First Affiliated Hospital of Nanchang Medical College), Nanchang, 330006 People’s Republic of China; 2 Department of Orthopedics SurgeryDonghu DistrictJiangxi Province, Jiangxi Provincial People’s Hospital, (The First Affiliated Hospital of Nanchang Medical College), 330006. No. 92 Aiguo Road, Nanchang, People’s Republic of China; 3 Department of Nursing Science, Jiangxi Provincial People’s Hospital, (The First Affiliated Hospital of Nanchang Medical College), Nanchang, 330006 People’s Republic of China

**Keywords:** Distal clavicular fracture, Tight-rope technique, Clavicular hook plate, Arthroscopic

## Abstract

**Background:**

The aim of the present study was to compare the clinical efficacy of arthroscopic-assisted fixation using the Tight-rope system and clavicular hook plate fixation in the treatment of Neer IIB distal clavicle fractures.

**Methods:**

We enrolled 48 consecutive patients with Neer IIB distal clavicle fractures who were treated at our institution from February 2016 and August 2020. These patients were divided into 2 groups based on the fixation method (16 cases with Tight-rope system and 32 cases with clavicular hook plate), and demographics and clinical characteristics of patients in different groups were compared.

**Results:**

All 48 patients had functional outcome scores of the affected shoulder available at a mean of 23.8 ± 5.1 months, and there was a statistically significant improvement in the constant score, American shoulder and elbow surgeons (ASES) score, visual analogue scale (VAS) score at the end of follow-up (*p* < 0.001 respectively). However, the smaller length of skin incision, less estimated blood loss and shorter hospital stay were detected in the Tight-rope technique group patients than those of clavicular hook plate group patients (*p* < 0.001, respectively). Furthermore, the constant score, ASES score and VAS score were significantly improved in the Tight-rope technique group patients than those of clavicular hook plate group patients (*p* < 0.05, respectively).

**Conclusions:**

Both Tight-rope technique and clavicular hook plate fixation can provide satisfactory clinical and radiological results in the treatment of distal clavicular Neer IIB fracture. However, arthroscopic-assisted fixation using the Tight-rope technique showed better results in terms of length of hospital stay, surgical trauma, clinical scores, and diagnose and treat concomitant glenohumeral pathologies.

**Levels of Evidence:**

III, Case–control study Retrospective comparative study.

## Introduction

Distal clavicle fractures run up nearly 20% of all clavicle fractures in human beings. Treatment of distal clavicle fractures remains a controversial topic for orthopaedic surgeons. In general, conservative treatments were recommended for nondisplaced fractures and surgical interventions were suggested for unstable fractures [1]. Neer’s type IIB fractures are associated with fracture and medial coracoclavicular ligament tear, leading to significant displacement and instability.The nonunion rate after conservative treatment is reported to be as high as 33%. therefore, most of the studies indicated that internal fixation surgery should be recommended for patients with such type of injury [2].

Recently, growing interest has focused on the fracture fixation for distal clavicle fractures. Numerous surgical techniques used for fixation, like using K-wire or hook plate for direct osteosynthesis, and using various suture materials or tendon grafts for indirect stabilization of the coracoclavicular ligament, were without a gold standard procedure [3]. Furthermore, more evidence suggested that the arthroscopic-assisted treatment of displaced distal clavicle fractures is superior to the open surgical techniques because it is a relatively minimally invasive procedure with fewer complications [4].

Arthroscopic assisted treatment of displaced distal clavicle fractures using Tight-rope system techniques had been reported in a few studies, and the current literature lacks studies to compare different stabilizing techniques with clinical outcome assessments [2–5]. The purpose of the present study was comparing the clinical and radiological outcome of the Tight-rope technique with the clavicular hook plate technique in the treatment of Neer’s type IIB displaced distal clavicle fractures. Also, we hypothesized that the new Tight-rope-fixation techniques lead to comparable or even better results than the clavicular hook plate technique.

## Methods

### Patient selection

This study was approved by our institutional research ethics committee. We enrolled 48 consecutive patients who were treated with a Tight-rope system or hook plate fixation for Neer IIB distal clavicle fractures at our institution from february 2016 and august 2020. All the patients were divided into 2 groups based on the fixation method: 16 patients (Tight-rope system group) were treated with arthroscopic assisted fixation using the Tight-rope system, and 32 patients (clavicular hook plate group) underwent fixation with clavicular hook plate.

**Fig. 1:**
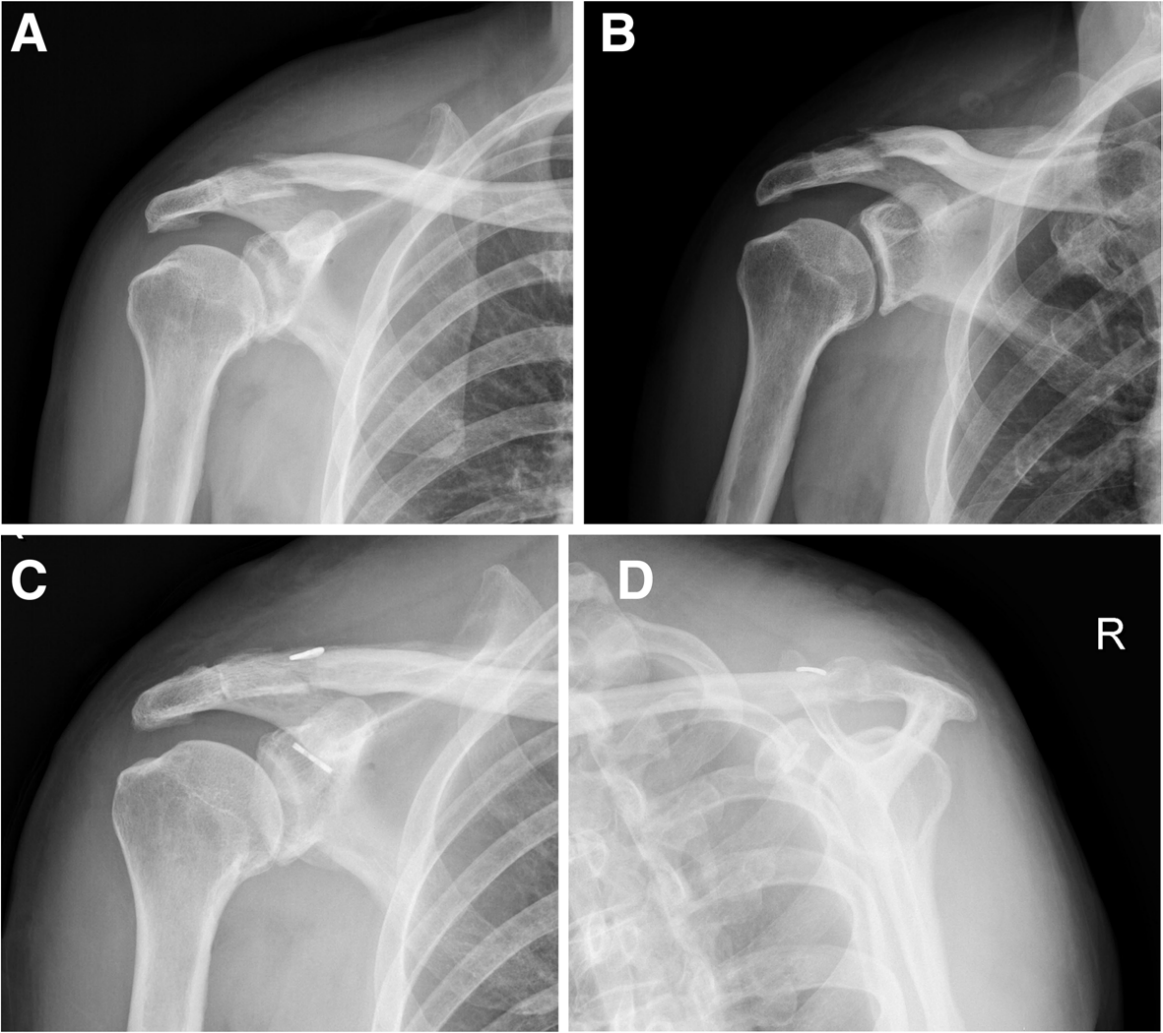
A 31-year-old female with a painful shoulder after falling, (**A**.**B**) preoperative radiograph showing Neer IIB distal clavicle fractures, (**C**.**D**) postoperative radiograph after treated with arthroscopic assisted fixation using Tight-rope technique

Diagnosis of patients with Neer IIB distal clavicle fractures primarily relies on clinical evidence, plain film (Fig. 1A and B), and computed tomography. Arthroscopic-assisted minimal invasive closed loop double endobutton technique with the Tight-rope system, and the arthroscopic evaluation and treatment of glenohumeral lesions were performed before internal fixation. Previous surgical history of the affected shoulder, other type of fracture, open fracture, those with additional upper extremity injury, and concomitant fracture around the affected shoulder were excluded from this study. Moreover, patients presenting with concomitant pathologies (such as significant psychiatric or neuromuscular) that could potentially preclude accurate evaluation were excluded from this study.

### Data collection

All the patients’ demographics and clinical characteristics were carefully recorded, including age, gender, side, and time interval from injury to surgery, mechanism of injury, comorbidities (diabetes mellitus, high blood pressure, alcohol consumption status and smoking status), and surgery related factors (treatment of distal clavicle fractures, the incision length, operation time, intraoperative blood loss, hospitalization time, Neer fracture type). Clinical outcomes include preoperative and postoperative constant score, American shoulder and elbow surgeons (ASES) score and visual analogue scale (VAS).

### Surgical techniques

#### Clavicular hook plate techniques

The patient is in the supine position and their affected shoulder is elevated at 30º under general anesthesia. An approximately 8 cm long curved skin incision was made at the distal end of the clavicle to expose the fracture site. After reduction, the clavicle was held with K-wires, then the clavicular hook plate was placed on the lateral clavicle and fixed with screws. The position of internal fixation was evaluated with intraoperative fluoroscopy, and then routine irrigation and closure of the wound were performed.

#### Tight-rope techniques

**Fig. 2:**
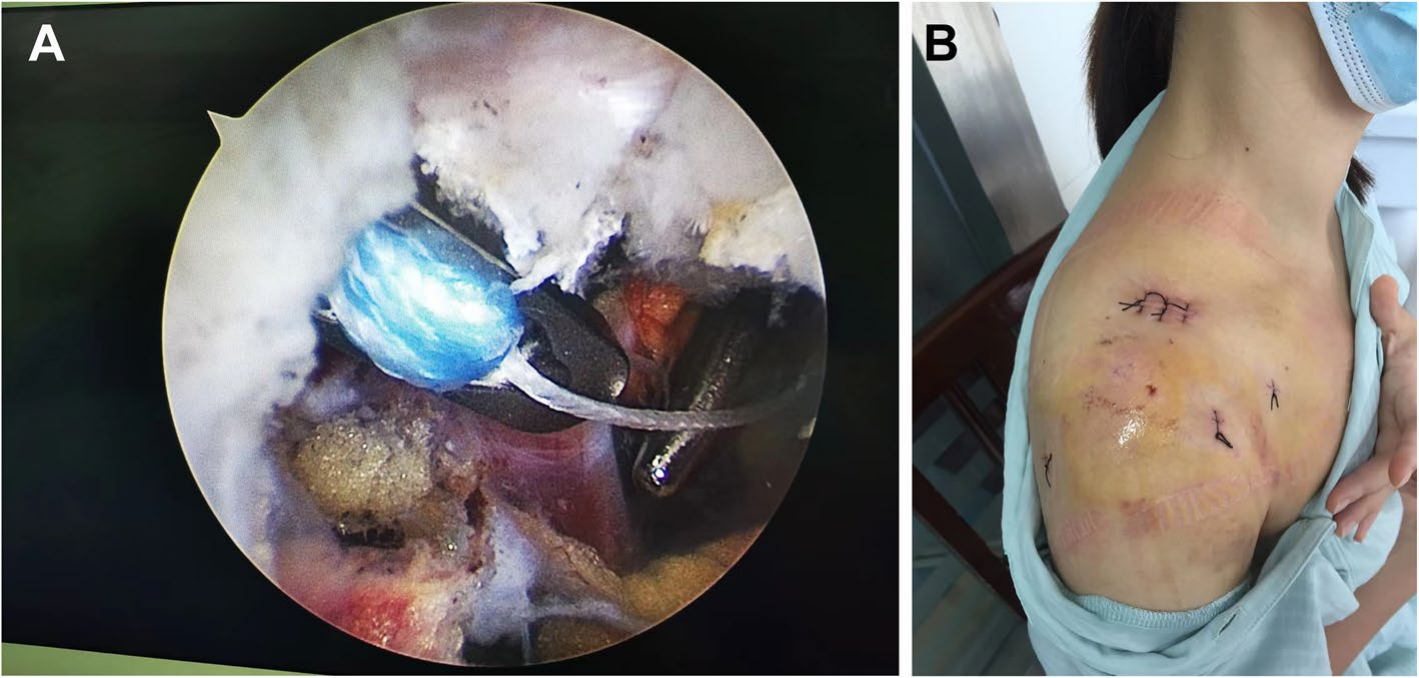
**A** The button of the Tight-rope system flipped on the inferior surface of the coracoid process. **B** Postoperative picture of the shoulder showing skin portals

Based on our previous research [6], the patient was placed in the beach-chair position under general anesthesia. The concomitant glenohumeral pathologies were evaluated and treated, and the base of the coracoid was exposed from intraarticular during arthroscopically. An approximately 2 cm incision was performed on the superior surface of the lateral clavicle to the coracoid, and then a 2.0 mm guide pin was advanced through the proximal clavicle fragment to the base of the coracoid. Subsequently, the Tight-rope system was inserted into both tunnels with the special guide system assisted under arthroscopic visualization control (Fig. 2A), and the fracture reduction was confirmed under fluoroscopy control. The Tight-rope system was tightened after satisfactory tension was achieved, and the standard closure of the incision site (Fig. 2B).

### Postoperative management

The bone healing of the clavicle was confirmed relies on plain film, all patients shoulder was protected with a sling for 6 weeks, the normal use of the arm for most activities of daily living was encouraged as tolerable, gradual full active range of motion exercises of the shoulder was started after 6 weeks, heavy strenuous activities were allowed after 3 months depending on the patient’s clinical situation.

### Outcome assessment

Physical examination and radiography of surgical patients were evaluated independently by two investigators, and imaging (Fig. 1C and D) and physical examination were performed regularly after surgery (at directly postoperative, 1, 3, 6, 12 months and yearly until final follow-up). Shoulder functional assessment was performed according to the constant score and ASES score (the higher scores indicate the greater function), and all the patients were evaluated pre-operatively as well as in post-operative follow-up period using 10- point VAS for knee pain (the higher scores indicate the greater pain).

### Statistical analysis

All analysis was performed by IBM SPSS Version 22 (SPSS Inc. Chicago IL). Continuous variables the two groups were compared using the Student’s t-test, and noncontinuous variables were assessed by the Chi-square test. Statistical significance was set as *P* value less than 0.05.

## Results

### Patient demographics

The patients’ demographics were showed in Table 1. The Tight-rope system group patients were treated with arthroscopic assisted fixation using the Tight-rope techniques, also, the concomitant glenohumeral pathologies were detected in 2 patients during arthroscopically surgical procedure (1 patient showed a full thickness rotator cuff tear and 1 patient had a Bankart lesion). In detail, there were no significant differences in demographic data, duration from injury to the surgery, injured side, comorbidities and mechanism of injury between the two surgical groups (*p* > 0.05, respectively). The mean follow-up was 22.2 ± 5.3 months in Tight-rope system group, and 24.5 ± 4.9 months in the clavicular hook plate group, with no significant difference for follow-up period was found among the two groups (*P* > 0.05).

**Table 1:** Patient demographics in different groups

Characteristic	TR group (16)	CH group (32)	*P*
Age (y)	37.2 ± 11.6	36.1 ± 8.4	0.651
Gender: male n (%)	13 (81.3%)	25 (78.1%)	1
Mechanism of injury			
Traffic accident	7	16	0.807
Falling	7	11	0.673
Other	2	5	0.602
Comorbidities			
Diabetes mellitus	2	4	1
High blood pressure	3	5	0.786
Smoking status	2	5	0.770
Alcohol consumption status	3	7	0.800
Postoperative complications			
Infection	0	1	0.333
Fixation failure	0	0	1
Nonunion	0	0	1
Injured side: right n (%)	9(56.3%)	13(37.5%)	0.217
Follow-up time (month)	22.2 ± 5.3	24.5 ± 4.9	0.149
Injury to surgery(d)	6.5 ± 2.6	6.9 ± 3.2	0.588

*TR* Tight-Rope, *CH* Clavicular hook plate

### Complications

There were no significant differences in postoperative complications among the two groups (*p* > 0.05; Table 1). Intraoperative and postsurgical complications, such as infection, fixation failure and nonunion, were not found in the Tight-rope system group, comparing 3.1% complications in the clavicular hook plate group (infection 1 patient).

### Functional and clinical relative factors results of the study groups

Functional and clinical relative factors in the different groups are revealed in Table 2. All 48 patients had functional outcome scores available at a mean of 23.8 ± 5.1 months, and there was a statistically significant improvement in the constant score, ASES score and VAS score at the end of follow-up (*p* < 0.001, respectively).

**Table 2:** Functional and clinical result of the study groups

Characteristic	TR group (16)	CH group (32)	*P*
Length of surgery (h)	63.8 ± 9.7	64.7 ± 7.4	0.729
Estimated blood loss (ml)	58.8 ± 8.9	96.9 ± 20.2	< 0.001
Length of skin incision (cm)	4.1 ± 0.8	8.1 ± 1.0	< 0.001
Preoperative VAS score	6.9 ± 1.5	6.9 ± 1.6	1
Preoperative constant score	29.2 ± 10.4	27.7 ± 7.8	0.622
Preoperative ASES score	30.1 ± 8.6	28.2 ± 6.4	0.406
Postoperative VAS score	1.1 ± 0.6 ^a^	1.6 ± 0.7 ^a^	0.036
Postoperative constant score	89.6 ± 4.0 ^a^	84.4 ± 8.2 ^a^	0.022
Postoperative ASES score	88.9 ± 3.5 ^a^	83.9 ± 8.2 ^a^	0.005
Length of postoperative hospital stay (d)	3.8 ± 0.8	6.1 ± 1.0	< 0.001

*TR* Tight-Rope, *CH* Clavicular hook plate, *ASES* American shoulder and elbow surgeons, *VAS* visual analogue scale

^a^ significant improvement in the constant score, ASES score and VAS score (*p* < 0.001, respectively)

Based on the analysis, there were no statistically significant differences in length of surgery, preoperative constant score, ASES score and VAS score among two groups (*p* > 0.05, respectively). However, there was a smaller length of skin incision, less estimated blood loss and shorter hospital stay for the Tight-rope technique group patients than for clavicular hook plate group patients (*p* < 0.001, respectively). Furthermore, the constant score (Tight-rope system group: 89.6 ± 4.0 vs. clavicular hook plate group: 84.4 ± 8.2), ASES score (88.9 ± 3.5 vs. 83.9 ± 8.2) and VAS score (1.1 ± 0.6 vs. 1.6 ± 0.7) were significantly improved in the Tight-rope technique group patients than those in the clavicular hook plate group patients (*p* < 0.05, respectively).

## Discussion

The main finding of the present study is that the outcome of the arthroscopic-assisted fixation using the Tight-rope system for the stabilization of type IIB clavicular fracture was superior to the results of the clavicular hook plate. Compared with the clavicular hook plate, the Tight Rope technique is advantageous for treating these patients because it is a relatively minimally invasive procedure with fewer complications and better clinical outcomes.

Numerous surgical techniques used for the fixation of distal clavicle fractures, like using a K-wire or hook plate for direct osteosynthesis and using various suture materials or tendon grafts for indirect stabilization of the coracoclavicular ligament, were without a gold standard procedure [7]. More evidence suggests that the arthroscopic assisted treatment of displaced distal clavicle fractures is superior to the open surgical techniques because it is relatively durable and minimally invasive procedures with fewer complications [8, 9], In addition, the Tight-rope system can self-propelled adjust the reconstructed length, which has been proven to resist higher loads [10]. In line with previous studies, the present study showed successful clinical and radiological outcomes results for the displaced distal clavicle fractures patients who were treated with arthroscopic assisted fixation using a Tight-rope system, with a statistically significant difference between the preoperative and postoperative constant score, ASES score and VAS score.

In a recent small case series study, Erdle et al. [11] investigated the clinical and radiological outcomes of both hook plate and locking plate osteosynthesis fixations and found that both internal fixation techniques were equally effective in the treatment of Neer IIB fractures. However, due to the widespread use of clavicular hook plates, various complications associated with hook plates are well known [12]. In a retrospective meta-analysis, Oh et al. [13] investigated the direct osteosynthesis treatment results of 425 distal clavicle fracture patients and found that the incidence of complication rate was 1.6% to 22%. However, evidence indicates that the all-arthroscopic approach is advantageous for treating these patients because it is a minimally invasive procedure that successfully reduced the risk of infection and avoids the need for a second procedure for implant extraction [14]. In our study, patients who underwent clavicular hook plate had a higher incidence of infection than those in the Tight-rope system group, however, there was no significant correlation between postoperative complications and the surgical techniques in our study. Furthermore, the smaller length of skin incision, less estimated blood loss and shorter hospital stay were detected in the Tight-rope technique group patients than in those of clavicular hook plate group patients.

Flinkkilä et al. [15] demonstrated that both hook plate and arthroscopic surgery with the Tight-rope system can obtain comparable medium-term radiological and clinical results. However, 40.7% patients treated with hook plate fixation experienced complications. In addition, internal fixation is associated with acromial fractures in patients with osteoporosis and this procedure results in the osteolysis of the acromion and subacromial impingement, leading to the necessity for removal of the hook plate after bony union [16]. Our previous research compared results in 112 acromioclavicular joint separations between open surgery by hook plate (84 cases) and arthroscopic surgery with the Tight-rope technique (28 cases), and showed successful medium-term radiological and clinical results for the patients who were treated with the Tight-rope technique [6]. Cho et al. [17] analyzed the clinical and radiological results of coracoclavicular stabilization using Tight-rope technique for Neer type IIB distal clavicle fractures, it was found that the Tight-rope system is an effective method for improvement of ASES scores (postoperative ASES scores were 88.6) at middle follow-up period. In line with previous studies, compared with patients of clavicular hook plate group, those patients of the Tight-rope group demonstrated statistically significant improvement in functional recovery at the end of follow up. Furthermore, our study showed an important advantage of the routine arthroscopy with this method allows detection and repair of the frequently associated glenohumeral joint lesions (rotator cuff tear and Bankart lesion).

Several limitations were also detected in this study. First, our current study is a single-center non-randomized retrospective study, and a relatively small number of distal clavicular fractures patients were treated with Tight-rope system may introduce bias into the results, however, most of the literatures on Neer IIb distal clavicle fractures is limited by small sample sizes, further another multicenter prospective randomized clinical trial with increased sample size is required to validate this conclusion. Second, our study does not fully show what the outcome results would be while performing arthroscopic surgery with Tight-rope technique alone without performing any other glenohumeral joint lesions surgery, the difference in outcomes could be related to the management of other pathology in 2 patients. Third, we just observed the physical examination and radiographically of operatively treated patients at a mean of 23.8 ± 5.1 months, therefore, a prospective study with long-term follow-up is necessary.

## Conclusions

In conclusion, both the Tight-rope technique and clavicular hook plate fixation can provide reliable clinical and radiological results in the treatment of distal clavicular Neer IIB fracture. However, Arthroscopic assisted fixation using the Tight-rope technique showed better results in the length of hospital stay, surgical trauma, clinical scores, and diagnosis and treatment concomitant glenohumeral pathologies.

## Data Availability

The datasets generated and/or analysed during the current study are not publicly available due to data containing information that could compromise research participant privacy/consent but are available from the corresponding author on reasonable request.

## Abbreviations

ASES: American shoulder and elbow surgeons
VAS: Visual analogue scale
